# Factors Associated With Repeat Emergency Department Visits for Low Back Pain

**DOI:** 10.7759/cureus.21906

**Published:** 2022-02-04

**Authors:** Martinus Megalla, Chinwe Ogedegbe, Angeline M Sanders, Nicole Cox, Thomas DiSanto, Haley Johnson, Michael Kelly, John D Koerner

**Affiliations:** 1 Department of Orthopedic Surgery, Hackensack Meridian School of Medicine, Nutley, USA; 2 Department of Emergency Medicine, Hackensack University Medical Center, Hackensack, USA; 3 Office of Clinical Research Administration, Hackensack University Medical Center, Hackensack, USA; 4 Department of Orthopedic Surgery, Hackensack University Medical Center, Hackensack, USA; 5 Department of Orthopaedic Surgery, Rothman Orthopaedic Institute, Paramus, USA

**Keywords:** nsaids, insurance, hospital readmission, opioids, low back pain

## Abstract

Background

Low back pain represents 2-3% of Emergency Department (ED) visits. In this study, we aimed to identify patient and treatment-related variables that contributed to repeat visits to the ED for low back pain within a 12-month period.

Methodology

We conducted a retrospective review of adult patients presenting to the ED of one hospital over a two-year period with the primary diagnosis of low back pain. The primary outcome included return to the ED within 12 months with the same complaint, and the secondary outcome included return to the ED within 30 days or six months.

Results

A total of 793 patients met the inclusion criteria. The rate of return to the ED with the same complaint within 30 days, six months, and 12 months of the first visit was 7%, 11%, and 14%, respectively. Patients who received opioids at discharge were more likely to return within 12 months (68% vs. 55%; p = 0.0075) and six months (68% vs. 56%; p = 0.0184) compared to those who did not receive opioids at discharge. Undergoing an X-ray decreased the odds of a 30-day return visit by 70% (p = 0.0067), and by 59% within 12 months (p = 0.0032). Receiving opioids at discharge also doubled the odds of return within 12 months (odds ratio = 2.030, p = 0.0183), while receiving nonsteroidal anti-inflammatory drugs (NSAIDs) reduced the odds by 60% (p = 0.0028).

Conclusions

Patients who received opioids at discharge were more likely to have a return visit for low back pain within six and 12 months. Patients who underwent X-rays at the index visit and were prescribed NSAIDs at discharge were less likely to return to the ED for low back pain.

## Introduction

Acute low back pain in the adult population is common, accounting for 2-3% of Emergency Department (ED) visits [1-3]. Identifying patients who need pain control and outpatient follow-up versus those who need a more extensive workup with advanced imaging can be difficult. Protocols and algorithms have been developed to treat adult patients efficiently and thoroughly with nontraumatic low back pain [4-6]. Nonetheless, patients frequently return to the ED with the same complaint within a short period.

One of the most common treatment methods for low back pain is the administration of opioids, which has been shown to be moderately effective in the short-term [7]. However, treatment with opioids may not be more effective than other medications, such as nonsteroidal anti-inflammatory drugs (NSAIDs), and has a greater risk of detrimental outcomes than other treatments [8]. Furthermore, opioid dependency can lead to a greater likelihood of repeat visits to the ED for patients with chronic pain [9]. Early prescriptions of opioids in the ED have been shown to lead to increased long-term use of opioids [10]. The opioid epidemic has become a significant issue in healthcare [11,12]. The dangers of opioids loom large as several studies have indicated the risk of addiction among patients who are prescribed opioids for pain management [13-15].

Many centers now attempt to provide alternative treatments for low back pain in the ED. However, the long-term effectiveness of such treatments is unclear [16]. Multidisciplinary modalities including physical therapy have been shown to be effective in lowering healthcare utilization and costs [17]. The goals of protocols for patients with nontraumatic low back pain are to treat patients effectively and efficiently while ruling out patients with “red-flag” symptoms, as well as providing appropriate follow-up. Among other benefits, implementing a protocol can reduce the rate of return to the ED for these patients with similar symptoms. However, identifying which patient variables and treatment methods contribute to repeat visits for low back pain is essential in creating a successful treatment program. Radiographs typically have a limited role in the nontraumatic setting [18], but patients may perceive X-rays as an important study in the evaluation of low back pain [19].

The goal of this study was to identify patient and treatment-related variables that contributed to repeat visits to the ED for low back pain within a 12-month period. We hypothesized that medications administered in the ED and prescribed at discharge as well as insurance status would contribute to patients returning to the ED within 12 months with the same complaints. These findings will be beneficial when modifying new protocols for adult patients presenting to the ED with low back pain.

## Materials and methods

We conducted a retrospective review of adult patients who presented to the ED for back pain at one hospital over a two-year period (January 1, 2013, to December 31, 2014). Patients were identified via a search in the Electronic Medical Record for a primary diagnosis of low back pain with or without sciatica. Inclusion criteria included a primary diagnosis of low back pain and age of >18 years. Institutional review board approval was obtained prior to the start of the study. Data were collected on the following variables: age, sex, body mass index (BMI), Charlson Comorbidity Index (CCI) [20], insurance status, opioids taken before admission, medications administered in the ED (steroids, opioids, NSAIDs, muscle relaxants), and medications prescribed at discharge, imaging studies performed (X-rays, CT, MRI), ED and hospital length of stay, procedures performed, whether follow-up was provided, and whether a spine consult was obtained. The primary outcome was a return visit within 12 months, and secondary outcomes were a return visit within 30 days and six months. Return visits were based on the index visit discharge date. For patients with multiple return visits, a return visit could serve as an index visit for the following ED visit.

Data were collected by trained research associates who were blinded to the study hypothesis using a standardized data collection form which was then stored in a centralized database. Data were monitored and evaluated for accuracy at various time points throughout data collection by a second researcher.

While age, CCI, BMI, and hospital length of stay were continuous variables, they were categorized for analysis due to violations of the linearity assumption [21] and to improve interpretability. Previous literature, percentiles, and adequate event rates to estimate effects were used in combination to create the following categories: Age (<40, 40-59, 60+ years) [22]; CCI (0, 1, ≥2) [23]; BMI (underweight/normal: 0-24.9 kg/m^2^, overweight 25-29.9 kg/m^2^, obese 30+ kg/m^2^); Length of ED stay (less than three hours, three to four hours, more than four hours); Length of hospital stay (less than three hours, three to four hours, more than four hours).

Descriptive statistics were calculated for patients as well as individual visits. The number and frequency (%) were reported. Univariate logistic mixed models with random intercepts to account for the correlation between visits for the same patient were run for the primary and secondary outcomes. We constructed multivariate logistic mixed models to determine independent risk factors for 30-day and 12-month return visits. All variables in the univariate models with p-values of <0.15 were included in the multivariate models. Because age and CCI were collinear, the final model for 12-month return visits only included age, which improved model fit based on Bayesian Information Criteria. Adjusted odds ratios (ORs) and 95% confidence intervals (CIs) were reported for the multivariate models. P-values of <0.05 were considered statistically significant, and the complete case approach for missing data was used. All statistical analyses were performed in SAS version 9.4 (SAS Institute Inc., Cary, NC, USA).

## Results

In total, 793 patients corresponding to 928 ED visits met the inclusion criteria. Patient characteristics are listed in Table 1.

**Table 1:** Characteristics of patients presenting to the ED with back pain. The following variables have missing data: BMI (n = 46, 5.80%) and insurance (n = 2, 0.25%). BMI: body mass index; CCI: Charlson Comorbidity Index; ED: Emergency Department

Covariate	Number (%)
Number of patients	793
Female	461 (58.13)
Age (years)	
<40	254 (32.03)
40–59	340 (42.88)
60+	199 (25.09)
BMI	
Underweight: <18.5 kg/m^2^	11 (1.39)
Normal: 18.5–24.9 kg/m^2^	186 (23.46)
Overweight: 25–29.9 kg/m^2^	270 (34.05)
Obese: 30+ kg/m^2^	280 (35.31)
Insurance	
Private insurance	310 (39.09)
Medicaid	64 (8.07)
Medicare	151 (19.04)
Other	266 (33.54)
CCI	
0	394 (49.68)
1	156 (19.67)
≥2	243 (30.64)

Most patients were females (58%), 32% were less than 40 years old, 43% were 40-59 years old, and 25% were 60 or above. Approximately half of the patients had a CCI score of zero, while 31% of patients had a CCI score of two or higher. The most common insurance type was private insurance (39%). Overall, 10% of patients had more than one ED visit, and the number of visits ranged from one to 16 during the two-year study period.

Characteristics of the 928 patient visits are presented in Table 2. Approximately one-third of patients were using opioids prior to their ED visit. While in the ED, NSAIDs were the most common drug administered (61% of visits), followed by oral opioids (41% of visits) and muscle relaxants (41% of visits). At discharge, opioids, muscle relaxants, and NSAIDs were all frequently prescribed (57%, 44%, and 42% of visits, respectively), while patients received steroids less often (12% of visits). X-rays were commonly performed (43% of visits), and patient follow-up was provided in three-fourths of the visits. Spine consultations were reported for 4% of visits, and procedures were conducted for 1% of visits. The ED return visit rates at 30 days, six months, and one year were approximately 7%, 11%, and 14%, respectively.

**Table 2:** Characteristics of ED back pain patient visits and univariate analysis of return visits at 30 days, six months, and 12 months. Note: Bold p-values indicate p-values of <0.05. The following variables have missing data: BMI (n = 46, 4.96%), insurance (n = 2, 0.22%), on narcotics prior to visit (n = 1, 0.11%), oral steroids in ED (n = 1, 0.11%), opioids at discharge (n = 3, 0.32%), steroids at discharge (n = 4, 0.43%), NSAIDs at discharge (n = 3, 0.32%), muscle relaxants at discharge (n = 3, 0.32%), procedure performed (n = 1, 0.11%), follow-up (n = 2, 0.22%), and spine consult (n = 2, 0.22%). ED: Emergency Department; BMI: body mass index; CCI: Charlson Comorbidity Index; NSAIDs: nonsteroidal anti-inflammatory drugs; IV: intravenous; CT: computed tomography; MRI: magnetic resonance imaging

	Full sample	Return to ED within 30 days			Return to ED within 6 months			Return to ED within 12 months		
Covariate		No	Yes	P-value	No	Yes	P-value	No	Yes	P-value
Number of visits	928	864 (93.10)	64 (6.90)		826 (89.01)	102 (10.99)		800 (86.21)	128 (13.79)	
Female	524 (56.47)	498 (57.64)	26 (40.63)	0.1967	479 (57.99)	45 (44.12)	0.3713	466 (58.25)	58 (45.31)	0.2507
Age (years)										
<40	275 (29.63)	268 (31.02)	7 (10.94)	0.1860	260 (31.48)	15 (14.71)	0.0452	254 (31.75)	21 (16.41)	0.0048
40–59 (ref)	434 (46.77)	388 (44.91)	46 (71.88)		362 (43.83)	72 (70.59)		344 (43.00)	90 (70.31)	
60+	219 (23.60)	208 (24.07)	11 (17.19)		204 (24.70)	15 (14.71)		202 (25.25)	17 (13.28)	
BMI										
Underweight/Normal: <25kg/m^2 ^(ref)	228 (24.57)	208 (25.43)	20 (31.25)	0.8681	201 (25.77)	27 (26.47)	0.7628	199 (26.39)	29 (22.66)	0.4450
Overweight: 25–29.9 kg/m^2^	324 (34.91)	301 (36.80)	23 (35.94)		287 (36.79)	37 (36.27)		274 (36.34)	50 (39.06)	
Obese: 30+ kg/m^2^	330 (35.56)	309 (37.78)	21 (32.81)		292 (37.44)	38 (37.25)		281 (37.27)	49 (38.28)	
Insurance										
Private insurance (ref)	336 (36.21)	321 (37.24)	15 (23.44)	0.3717	314 (38.11)	22 (21.57)	0.0532	310 (38.85)	26 (20.31)	0.0061
Medicaid	95 (10.24)	83 (9.63)	12 (18.75)		74 (8.98)	21 (20.59)		68 (8.52)	27 (21.09)	
Medicare	172 (18.53)	162 (18.79)	10 (15.63)		155 (18.81)	17 (16.67)		152 (19.05)	20 (15.63)	
Other	323 (34.81)	296 (34.34)	27 (42.19)		281 (34.10)	42 (41.18)		268 (33.58)	55 (42.97)	
CCI										
0 (ref)	453 (48.81)	424 (49.07)	29 (45.31)	0.7310	406 (49.15)	47 (46.08)	0.1019	397 (49.63)	56 (43.75)	0.0193
1	201 (21.66)	184 (21.30)	17 (26.56)		168 (20.34)	33 (32.35)		158 (19.75)	43 (33.59)	
≥2	274 (29.53)	256 (29.63)	18 (28.13)		252 (30.51)	22 (21.57)		245 (30.63)	29 (22.66)	
On narcotics prior to visit	290 (31.25)	259 (30.01)	31 (48.44)	0.4537	244 (29.58)	46 (45.10)	0.6784	233 (29.16)	57 (44.53)	0.2817
Received oral steroids in ED	78 (8.41)	75 (8.69)	3 (4.69)	0.2660	69 (8.36)	9 (8.82)	0.7124	65 (8.14)	13 (10.16)	0.2960
Received NSAIDs in ED	570 (61.42)	540 (62.50)	30 (46.88)	0.1067	517 (62.59)	53 (51.96)	0.3302	500 (62.50)	70 (54.69)	0.6707
Received IV opioids in ED	170 (18.32)	149 (17.25)	21 (32.81)	0.1147	144 (17.43)	26 (25.49)	0.6882	140 (17.50)	30 (23.44)	0.7417
Received oral opioids in ED	381 (41.06)	355 (41.09)	26 (40.63)	0.8479	335 (40.56)	46 (45.10)	0.5609	325 (40.63)	56 (43.75)	0.8813
Received muscle relaxants in ED	380 (40.95)	358 (41.44)	22 (34.38)	0.4250	340 (41.16)	40 (39.22)	0.9810	327 (40.88)	53 (41.41)	0.7208
Received IV steroids in ED	22 (2.37)	19 (2.20)	3 (4.69)	0.8756	18 (2.18)	4 (3.92)	0.6270	18 (2.25)	4 (3.13)	0.9300
Received opioids at discharge	526 (56.68)	484 (56.21)	42 (65.63)	0.1380	457 (55.53)	69 (67.65)	0.0184	439 (55.08)	87 (67.97)	0.0075
Received steroids at discharge	108 (11.64)	98 (11.40)	10 (15.63)	0.4485	95 (11.56)	13 (12.75)	0.8022	90 (11.31)	18 (14.06)	0.3852
Received NSAIDs at discharge	387 (41.70)	371 (43.09)	16 (25.00)	0.6534	362 (43.99)	25 (24.51)	0.1496	356 (44.67)	31 (24.22)	0.0240
Received muscle relaxants at discharge	409 (44.07)	384 (44.60)	25 (39.06)	0.9603	370 (44.96)	39 (38.24)	0.6271	359 (45.04)	50 (39.06)	0.5204
X-ray performed	397 (42.78)	385 (44.56)	12 (18.75)	0.0104	375 (45.40)	22 (21.57)	0.0029	366 (45.75)	31 (24.22)	0.0037
CT performed	182 (19.61)	174 (20.14)	8 (12.50)	0.2636	169 (20.46)	13 (12.75)	0.2540	164 (20.50)	18 (14.06)	0.3501
MRI performed	40 (4.31)	38 (4.40)	2 (3.13)	0.9460	35 (4.24)	5 (4.90)	0.5219	34 (4.25)	6 (4.69)	0.6853
Procedure performed	12 (1.29)	12 (1.39)	0 (0.00)	0.9781	12 (1.45)	0 (0.00)	0.9847	11 (1.38)	1 (0.78)	0.9142
Follow-up	701 (75.54)	646 (74.86)	55 (87.30)	0.0684	619 (75.03)	82 (81.19)	0.2570	598 (74.84)	103 (81.10)	0.2818
Spine consult	38 (4.09)	34 (3.94)	4 (6.35)	0.2718	32 (3.88)	6 (5.94)	0.4850	31 (3.88)	7 (5.51)	0.7263
Length of ED stay (hours)										
<3	361 (38.90)	337 (39.00)	24 (37.50)	0.9702	316 (38.26)	45 (44.12)	0.3032	304 (38.00)	57 (44.53)	0.2640
3–3.9 (ref)	226 (24.35)	211 (24.42)	15 (23.44)		205 (24.82)	21 (20.59)		199 (24.88)	27 (21.09)	
4+	341 (36.75)	316 (36.57)	25 (39.06)		305 (36.92)	36 (35.29)		297 (37.13)	44 (34.38)	
Length of hospital stay (hours)										
<3	352 (37.93)	328 (37.96)	24 (37.50)	0.9101	309 (37.41)	43 (42.16)	0.3641	298 (37.25)	54 (42.19)	0.4161
3–3.9 (ref)	222 (23.92)	207 (23.96)	15 (23.44)		202 (24.46)	20 (19.61)		196 (24.50)	26 (20.31)	
4+	354 (38.15)	329 (38.08)	25 (39.06)		315 (38.14)	39 (38.24)		306 (38.25)	48 (37.50)	

Univariable analyses

Results from the univariable analyses of characteristics associated with return visits at 30 days, six months, and 12 months are presented in Table 2.

At all three time points, receiving an X-ray at the index visit was inversely associated with an ED return visit for back pain. Patients receiving an X-ray at the index visit were less likely to return to the ER within 30 days (19% vs. 45%; p = 0.0104), six months (22% vs. 45%; p = 0.0029), and 12 months (24% vs. 46%; p = 0.0037).

Those who received opioids at discharge were more likely to visit the ED within six months of the index visit (68% vs. 56%; p = 0.0184) and within 12 months (68% vs. 55%; p = 0.0075).

Middle-aged patients between the ages of 40 and 59 were more likely than their younger and older counterparts to return to the ED within six months (p = 0.0452) and 12 months (p = 0.0048). Receiving NSAIDs at discharge had an inverse relationship with a return visit within 12 months (24% vs. 45%; p = 0.0240).

Insurance through Medicaid increased return visit risk relative to private insurance (p = 0.0061), and patients with a CCI of one were more likely to return than those with a CCI of zero (p = 0.0193).

Multivariable analyses

Multivariable analyses were performed to further identify independent risk factors for 30-day and 12-month return visits. Based on the univariate analyses, X-ray, follow-up, NSAIDs in ED, intravenous opioids in ED, and opioids at discharge were included in the multivariate model for 30-day returns (Table 3). Receiving an X-ray was the only independent risk factor, decreasing return risk by 70% (p = 0.0067).

**Table 3:** Multivariate model for factors associated with ED return within 30 days of the previous visit. Note: Variables with p-values of <0.15 in the univariate analyses were included in the multivariate model. Bolded p-values indicate p-values of <0.05. ED: Emergency Department; LCL: lower confidence limit; UCL: upper confidence limit; NSAIDs: nonsteroidal anti-inflammatory drugs; IV: intravenous

		95% confidence interval		
Covariate	Odds ratio	LCL	UCL	P-value
X-ray performed	0.304	0.129	0.714	0.0067
Follow-up	2.588	0.876	7.645	0.0847
Received NSAIDs in ED	0.550	0.261	1.157	0.1143
Received IV opioids in ED	2.027	0.812	5.058	0.1289
Received opioids at discharge	1.565	0.707	3.460	0.2665

Variables included in the final model for 12-month return visit included age, insurance, opioids and NSAIDs at discharge, and X-ray (Table 4). Compared to middle-aged patients between the ages of 40 and 59, younger and older age decreased return risk by 56% (p = 0.0292) and 69% (p = 0.0194), respectively. Those insured through Medicaid were 4.7 times more likely to have 12-month return visits compared to patients privately insured (p = 0.0016). Receiving opioids at discharge doubled the odds of 12-month return visits for back pain (OR = 2.030, 95% CI: [1.130, 3.647]; p = 0.0183) while receiving NSAIDs at discharge and having X-rays performed at the index visit decreased the odds by approximately 60% (p = 0.0028 and p = 0.0032, respectively).

**Table 4:** Multivariate model for factors associated with ED return within 12 months of the previous visit. Note: Variables with p-values of <0.15 in the univariate analyses were included in the multivariate model. CCI was excluded from the multivariate model due to high collinearity with age. Bolded p-values indicate p-values of <0.05. ED: Emergency Department; LCL: lower confidence limit; UCL: upper confidence limit; NSAIDs: nonsteroidal anti-inflammatory drugs; CCI: Charlson Comorbidity Index

		95% confidence interval		
Covariate	Odds Ratio	LCL	UCL	P-value
Age (years)				
<40	0.444	0.214	0.920	0.0292
40–59 (ref)				
60+	0.314	0.119	0.827	0.0194
Insurance				
Private insurance (ref)				
Medicaid	4.748	1.827	12.341	0.0016
Medicare	2.278	0.813	6.382	0.1162
Other	2.884	1.358	6.127	0.0062
Received opioids at discharge	2.030	1.130	3.647	0.0183
Received NSAIDs at discharge	0.398	0.219	0.725	0.0028
X-ray performed	0.413	0.230	0.739	0.0032

## Discussion

The treatment of patients presenting to the ED with low back pain can be challenging. After screening for “red flag” signs and symptoms, obtaining adequate pain relief and appropriate follow-up are necessary. Although opioids can be an effective treatment for low back pain, the potential side effects and the opioid epidemic have called into question their role in pain management. Our study has demonstrated that receiving opioids on discharge can contribute to the likelihood of patients returning to the ED for repeat treatment for low back pain. Receiving NSAIDs on discharge was associated with a decreased likelihood for return visits at 12 months, suggesting that NSAIDs can provide adequate pain relief for patients with low back pain. However, patients who were prescribed opioids were likely assessed as having more severe pain. Therefore, return visits to the ED may not have been a result of being prescribed opioids, but rather the fact that they had more severe or intractable pain. Alternatively, patients with substance use disorders may only return to the ED if they expect to be prescribed opioids on discharge, although we did not directly assess this in our study. These findings should be interpreted in the context of the location of our hospital and the availability of alternative EDs, which can contribute to lower rates of repeat visits because patients may choose to visit a different ED rather than return to the same one. Prescribing the appropriate medications on discharge is a difficult decision. Providers need to provide adequate pain relief, without overprescribing pain medications. Many states have also developed medication guidelines and databases that limit first-time opioid prescriptions.

Our rate of patients undergoing imaging (43%) is similar to previous studies which range from 28% to 56% [24,25]. The high rate of imaging observed in this study can be explained by a lack of differentiation between musculoskeletal back pain and radicular or sciatic nerve pain. Furthermore, protocols have been developed since this study period to reduce unnecessary imaging and follow guidelines more closely. A meta-analysis of six randomized control trials (1,804 patients) found no significant difference in short-term or long-term outcomes in patients receiving lumbar imaging for low back pain versus those receiving usual care without imaging [26]. These outcomes were primarily related to pain and function and did not assess return visits to an ED. We found that receiving an X-ray at the index visit was consistently associated with a decreased likelihood of return visits at 30 days, six months, and 12 months. It is possible that obtaining imaging gives patients more confidence and potentially reduces repeat visits. However, we did not evaluate whether the findings of these radiographs had any impact on treatment.

We found that oral opioids were administered at a rate of 41% in the ED. Interestingly, this rate of opioid administration is almost identical to the 42% rate reported in a similar study [25]. Overall, 57% of patients were prescribed opioids on discharge, which is also consistent with findings from a similar study [3]. Chronic pain management protocols have been studied for ED visits and have demonstrated decreased opioid administration in both the ED and upon discharge [27].

Evidence-based models for the treatment of low back pain are currently being studied and will hopefully reduce unnecessary tests, identify the most effective treatments, and ultimately improve patient outcomes [28]. In addition, nonmedical management such as self-directed exercises may help provide functional improvement in some patients with low back pain [29]. Minimizing the burden on the healthcare system is especially important during the coronavirus disease 2019 pandemic. A study in Italy found that visits for low back pain reduced by more than 87% in March-April 2020 compared to 2019, prompting the question of how many of these visits truly required immediate intervention in the ED [30]. Given these findings, it is increasingly important to develop ways to alleviate the stress on our EDs by finding ways to prevent unnecessary visits. One possible solution is implementing telehealth, which may help in the management of patients with various conditions, including low back pain [31].

Within our patient population, there was no difference in returning within 30 days for patients with private insurance versus Medicaid. However, patients with Medicaid were more likely to return within six months and had a higher odds of return visits within 12 months. Future studies should more thoroughly follow patients after discharge and try to identify obstacles that may preclude prompt primary care or specialist follow-up.

Limitations

The primary limitation of this study was its retrospective nature. Prospective data were not obtained which limits the ability to determine if there is any causal relationship between any of the associations that were observed. The patients included do not reflect a truly randomized sample but include all patients presenting to a singular institution within the given timeframe. The data were dependent on patient charts, which are subject to coding errors. However, patient data were collected by multiple individuals to reduce error in chart abstraction via double data entry. Furthermore, charts were reviewed for accuracy and consistency. There was no confirmation that opioids prescribed upon discharge were filled. Data on patient race and ethnicity were not obtained for this study but may be helpful to assess any possible relationship with the studied variables.

The data in this study is from 2013-2014 and should be interpreted with caution as various changes have been made since that time. For example, wisely choosing campaigns have emphasized that imaging is rarely needed for acute low back pain and should be used sparingly, resulting in improved imaging stewardship in the ED [32]. Additionally, providers have become increasingly diligent regarding the prescription of opioids, and rates of opioid prescriptions in the ED have declined significantly [33].

## Conclusions

In summary, receiving opioids on discharge may contribute to a return to the ED within six months and 12 months for low back pain. Furthermore, patients receiving NSAIDs at discharge and an X-ray at the index visit may be less likely to return to the ED for low back pain. Insurance status may also contribute to return visits for low back pain within six and 12 months.
